# Prognosis and immune features of pyroptosis-related RNA patterns in low-grade glioma

**DOI:** 10.3389/fonc.2022.1015850

**Published:** 2022-12-20

**Authors:** Hanzhang Liu, Tao Tao

**Affiliations:** ^1^ Morphology Laboratory, Medical College of Nantong University, Nantong, Jiangsu, China; ^2^ Department of Clinical Medicine, Ningbo College of Health Science, Ningbo, Zhejiang, China

**Keywords:** LGG, pyroptosis, prognostic model, bioinformatics, immunity

## Abstract

**Purpose:**

Low-grade gliomas (LGG), which are malignant primary brain tumors, are more prevalent in young adults. Pyroptosis, an inflammatory form of programmed cell death, has been shown in recent years to be directly associated with tumor growth and tumor microenvironment (TME). However, the correlation between LGG and pyroptosis remained to be explored. In this research, we explored pyroptosis-related gene expression patterns and their prognostic significance based on transcriptome profiles and clinical data in LGG.

**Methods:**

We identified 31 pyroptosis-related genes differentially expressed at the mRNA level between the data of LGG patients from TCGA and the data of normal brain tissues from GTEx. Univariate Cox regression analysis was used to screen 16 differentially expressed genes (DEGs) based on survival data. Next, the prognostic model was established using LASSO Cox regression, which divided LGG patients into high- and low- risk subgroups and showed an independent prognostic value for overall survival (OS) combined with clinical factors in the CGGA test cohort. Pyroptosis and immune cells were correlated through the CIBERSORT R package and the TIMER database.

**Results:**

Based on the analyses of 523 LGG and 1152 normal tissues, nine significant differential genes were identified. The AUC remained at about 0.74 when combined with the risk score and clinical factors. Enrichment analyses revealed that DEGs were mainly enriched in cytokine-cytokine receptor interactions, immune response and chemokine signaling pathways. Immune cell enrichment analysis demonstrated that scores for most immune cell types differed significantly between the high-and low-risk groups, and further infiltrating analysis showed obvious differences between these two risk subgroups.

**Conclusion:**

Pyroptosis-related genes play a pivotal role in LGG and are associated with tumor immunity, which may be beneficial to the prognosis and immunotherapy of LGG.

## Introduction

According to the World Health Organization (WHO), low-grade gliomas (LGG) account for about 20% of gliomas and are often diagnosed in young patients (1, 2). While most LGGs are inactive in patients, some cases rapidly deteriorate to neurological disorders and death in the short term (3). Based on prior clinical experience, an accurate and sensitive prognostic model may serve as the foundation for more effective diagnostic and therapeutic approaches.

According to the WHO classification guide, many molecular biomarkers are used to detect tumor occurrence, proliferation, infiltration and migration. These biomarkers provide clues by which to assess the comprehensive status of gliomas and then formulate personalized precision treatment (4). Previous studies on the biomarkers of glioma mainly focused on epidermal growth factor receptor (EFGR) amplification, chromosome 1p/19q deletion, O6-methylguanine DNA methyltransferase (MGMT) promoter methylation, and isocitrate dehydrogenase (IDH) mutations (5, 6). The literature confirms many studies with an appreciable quantity of reporting on prognostic models related to LGG, such as those involving metabolism (7), autophagy (8), ferroptosis (9), cuproptosis (10), and so on. While such markers and models are present in a wide variety of gliomas, they are insufficient for predicting the prognosis of LGG. Even though there have been many studies on prognostic models for LGG, no unified prognostic models have been applied in clinical practice. The complexity of glioma development and biological pathways call for the discovery of novel and specific LGG biomarkers.

Studies on malignancies of the nervous system have consistently highlighted the phenomena of programmed cell death (PCD) (11, 12). First, the formation of nerve tissue depends on the regulation of PCD. Abnormal death of nerve cells can promote tumor growth (11, 12). Second, tumor cells can resist or escape from cell-killing effects mediated by immune cells through a variety of adaptive mechanisms (13, 14). Thus, the detection and regulation of PCD performed an essential function in the diagnosis and treatment of nervous system tumors. Newly discovered close relationships among different kinds of tumors have made pyroptosis a hotspot as a new kind of PCD. Inflammasomes have been found in a large number of tumor cells, which activate the caspase pathways to initiate pyroptosis. Furthermore, pyroptosis can regulate the TME through proinflammatory effects to play a dual role in carcinomatosis and cancer promotion. This all indicates the potential of pyroptosis as a new biomarker of nervous system tumors (15, 16). Li et al. have constructed a prognostic model of glioblastoma (GBM, grade IV glioma) based on pyroptosis-related genes to provide accurate one, three, and five years overall survival rates (OS) of malignant gliomas (17). However, the indicators of LGG patients are still unclear, making it worthwhile to investigate the role of pyroptosis-related genes. Therefore, we developed a risk model for pyrolysis-related genes, which provides a new perspective for improving the prognosis and treatment of LGG.

## Material and methods

### Datasets

The transcriptome profile and clinical data of LGG patients were obtained from The Cancer Genome Atlas (18) (TCGA, https://www.tcga.org/) database and the Chinese Glioma Genome Atlas Project (19) (CGGA, http://www.cgga.org.cn/, **Supplementary Table 1**). Transcriptome profiles of 1152 normal brain tissues were collected from the Genotype-Tissue Expression Project (20) (GTEx, https://xenabrowser.net/) database. We acquired 523 low-grade gliomas in the TCGA database and 422 patients in the CGGA database to construct a validation set.

### DEGs identification

The gene expression data were normalized in all sets to fragments per thousand base million (FPKM) (21). The differentially expressed genes (DEGs) with P< 0.05 were identified by using the ‘limma’ R package (22). There are 33 pyroptosis-related genes were retrieved in Pubmed with ‘pyroptosis’ as the key word (23–25). In addition, a protein interaction network (PPI) was construct by using an interactive gene/protein search tool (String, http://www.string-db.org/) to analyze the correlation among DEGs. A Pearson correlation analysis diagram was drawn among DEGs.

### Development and validation of prognostic models for pyroptosis-related gene

In the training set, univariate COX regression analysis was adopted to determine the correlation between DEGs and overall survival (OS) to evaluate the prognostic value of DEGs in LGGs with the truncation condition as P< 0.05 and HR unequal to 1 (26). Next, Lasso were used to adjust COX proportional hazard regression to avoid overfitting (27). The above operations were performed through ‘glmnet’ R package, and penalty parameter λ was determined through minimum criterion (28). The prognostic model was constructed based on multivariate COX regression analysis (29). Based on the median risk score, LGG patients were assigned into low-risk group and high-risk group. The risk score formula following: risk score = $\sum_{i}^{n}$ Xi ×Yi, where n is the number of surviving genes after Lasso regression, Y is gene expression level, and X is coefficients. The OS time between subgroups was compared by using Kaplan-Meier analysis. The 3-year and 5-year ROC analyses were performed using the “survivalroc” R package and the area under the curve (AUC) was calculated. We used CGGA data as validation set to calculate the risk score. The Kaplan-Meier curves and ROC (Receiver Operating Characteristic) were plotted.

### Analysis of functional enrichment

The GO (Gene Ontology) (30) and KEGG (Kyoto Encyclopedia of Genes and Genomes) enrichment analysis (31) were performed by using ‘ClusterProfiler’ R package based on the difference between risk subgroups (|log2FC|≥mean(|log2FC|) + 4*sd(|logFC|);P<0.05).

### Estimation of tumor−infiltrating immune cells

According to the transcription profile of LGGs in TCGA, the relative proportion of immune infiltrating cells in all tumor samples was calculated by using cell type identification analysis in the ‘CIBERSORT’ R package (32, 33). We used the Wilcoxon rank-sum test to evaluate differences in immune cell infiltration levels between risk subgroups.

### TIMER database and GDSC database

The TIMER database provided a reliable estimate of the level of immune infiltration for tumor-immune interactions (34). The level of tumor immune infiltration and the correlation between gene expression were calculated by using ‘GENE’ module of the TIMER database (35). We screened a wide range of drugs from the GDSC database (36) and calculated the IC50 value of drugs using the pRRophetic algorithm (37) for patients with LGG.

### Quantitative real-time polymerase chain reaction

The HEB (human normal glial cell line) was obtained from Mingzhoubio (Ningbo, China) and was cultured H-DMEM medium with 10% FBS (fetal bovine serum). Human glioma cell lines, including U251 and U87, were obtained from the ATCC (American Type Culture Collection; Manassas, VA, USA) and were cultured in Roswell Park Memorial Institute (RPMI)-1640 with 10% fetal bovine serum (FBS). Total RNA from the cell lines was extracted by applying RNA simple Total RNA Kit (Tiangen, China). Subsequently, Total RNA was reverse transcribed with PrimeScript RT reagent kit (Takara, Otsu, Japan) to obtain cDNA. Using 2 μL cDNA and SYBR Premix Ex Taq (Takara, Otsu, Japan) and primers, the expression of the target genes was determined using the Biosystems StepOne Plus real-time PCR system (Life Technologies, Grand Island, NY, USA). The Primers of the target genes were obtained from Sangon Biotechnology (Shanghai, China), and the sequences are shown in **Supplementary Table 2**.

### Statistical analysis

Normally distributed variables were compared using Student’s t-test. Wilcoxon test was used to compare non-normally distributed data. Utilizing the Survminer package from R, we estimated the OS status between the two subgroups by Cox regression analysis and Kaplan–Meier curves. Ggplot2 package from R were used to plot the figures, thus visualizing our data. Test level was set at both sides α =0. 05, P < 0.05 was considered statistically significant unless otherwise specified.

## Results

**Figure 1:**
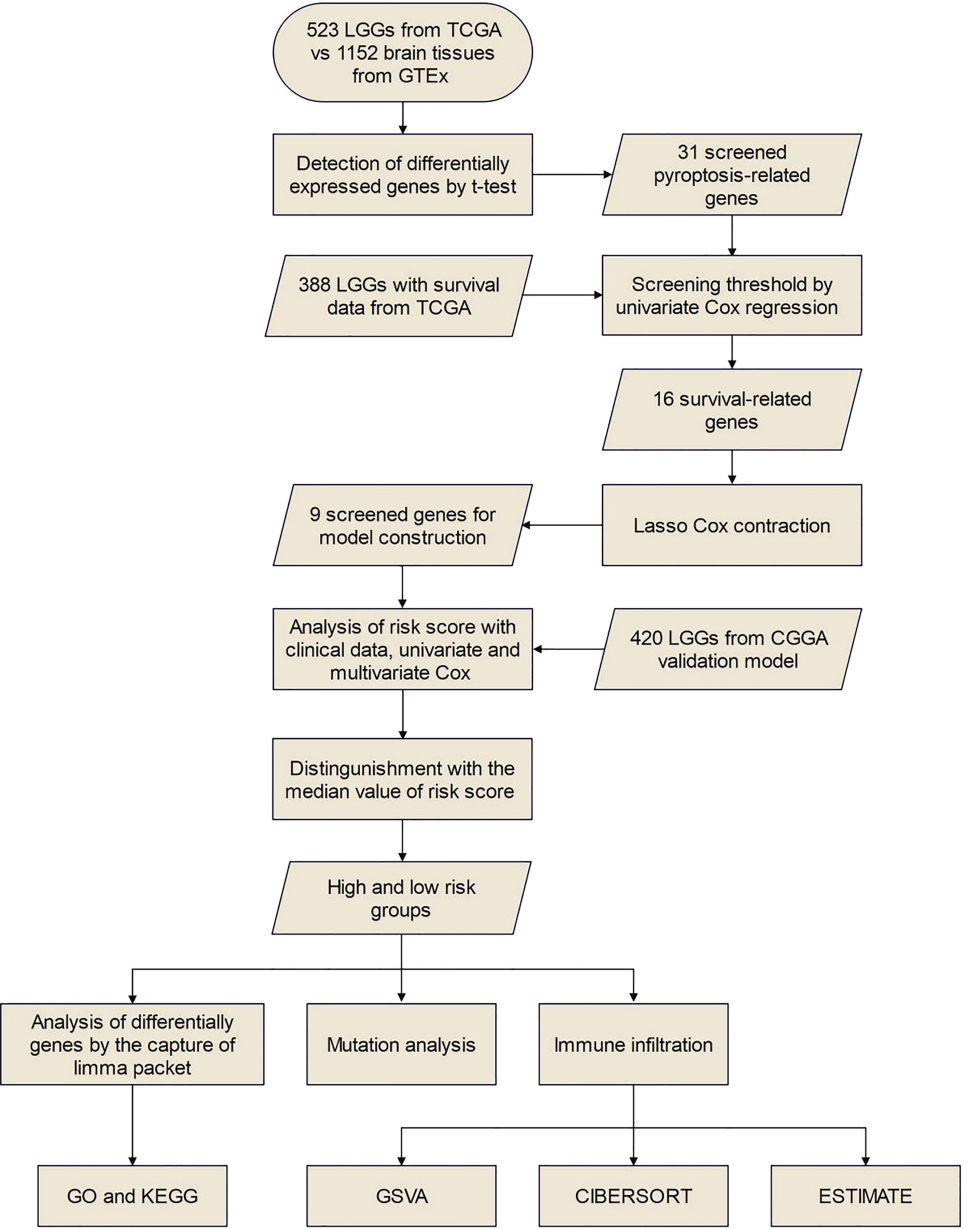
Specific workflow diagram of data analysis.

The flow chart of this study is shown in **Figure 1**.

### Identification of DEGs between tumor and normal tissues

**Figure 2:**
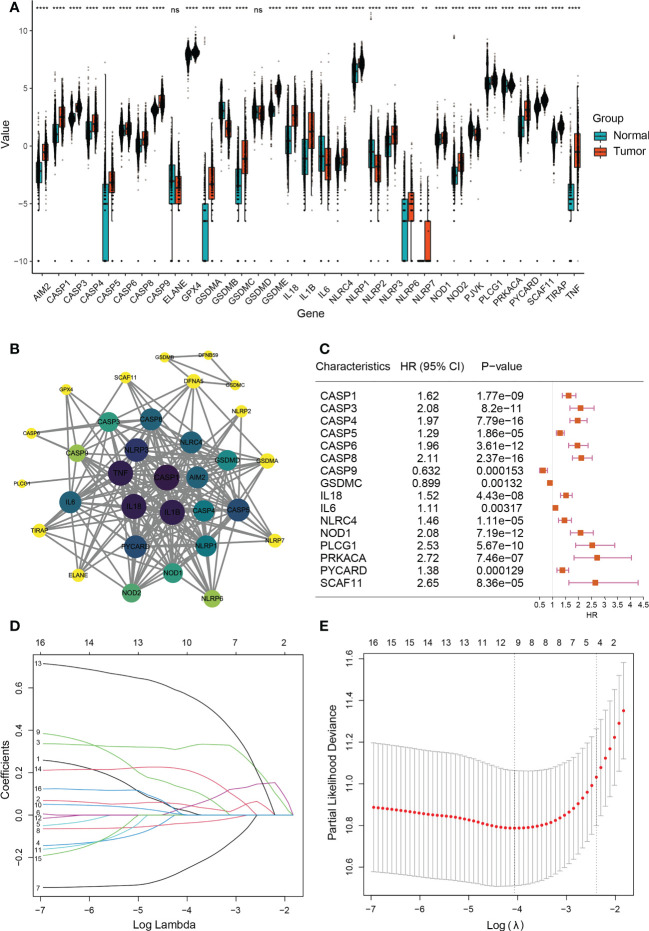
Expressions and screening of pyroptosis-related genes in low-grade glioma. **(A)** Box plot demonstrating 31 differentially expressed pyroptosis-related genes between normal tissue (n=1152) and lower-grade glioma (n=523). **P< 0.01; ∗∗∗∗P< 0.0001, ns: not significant. The green box shows normal tissue, and the red box shows tumor tissue. **(B)** PPI network showing the interactions of DEGs (interaction score=0.9). The darker the color of a node in a PPI network, the closer its connection to other nodes. **(C)** Univariate COX analysis of 16 DEGs (CASP1, CASP3, CASP4, CASP5, CASP6, CASP8, CASP9, GSDMC, IL18, IL6, NLRC4, NOD1, PLCG1, PRKACA, PYCARD, and SCAF11). P<0.05; HR, Hazard Ratio. **(D)** Cross-validation for tuning parameter selection in LASSO regression. **(E)** LASSO analysis of 9 prognostic pyroptosis-related genes.

First, we compared 33 pyroptosis-related genes in the pooled GTEx (Genotype-Tissue Expression) and TCGA (The Cancer Genome Atlas) data from 523 LGG and 1152 normal tissues and then identified 31 differentially expressed genes (DEGs) under the condition of P<0.05 (**Figure 2A** and **Supplementary Table 3**) besides ELANE and GSDMD. Among these DEGs, upregulated of 27 genes, while downregulated of 4 genes, in LGG tissues. The expression levels of DEGs were presented as heatmaps in **Figure S1**. We then constructed protein interaction networks (PPI) to investigate the interactions of DEGs (**Figure 2B**). As a result of the analysis, most DEGs showed obvious positive correlations, among which CASP4 and SCAF11 were significantly positively correlated (Cor = 0.87), while PRKACA was not connected to other DEGs.

### Development of a prognostic gene model in the training and testing sets

Next, A total of 388 LGG patients were matched based on their survival data. We used univariate Cox regression for preliminary analysis of DEGs, and 16 survival-related genes were screened at P<0.05 (**Figure 2C**). According to the optimum Λ value (**Figures 2D, E**), a prognostic model of 9 genes (CASP3, CASP4, CASP8, CASP9, GSDMC, IL18, IL6, PLCG1 and PRKACA) was construct by using Least absolute shrinkage and selection operator (LASSO) Cox regression analysis, thus calculating the risk score for each LGG patient.

**Figure 3:**
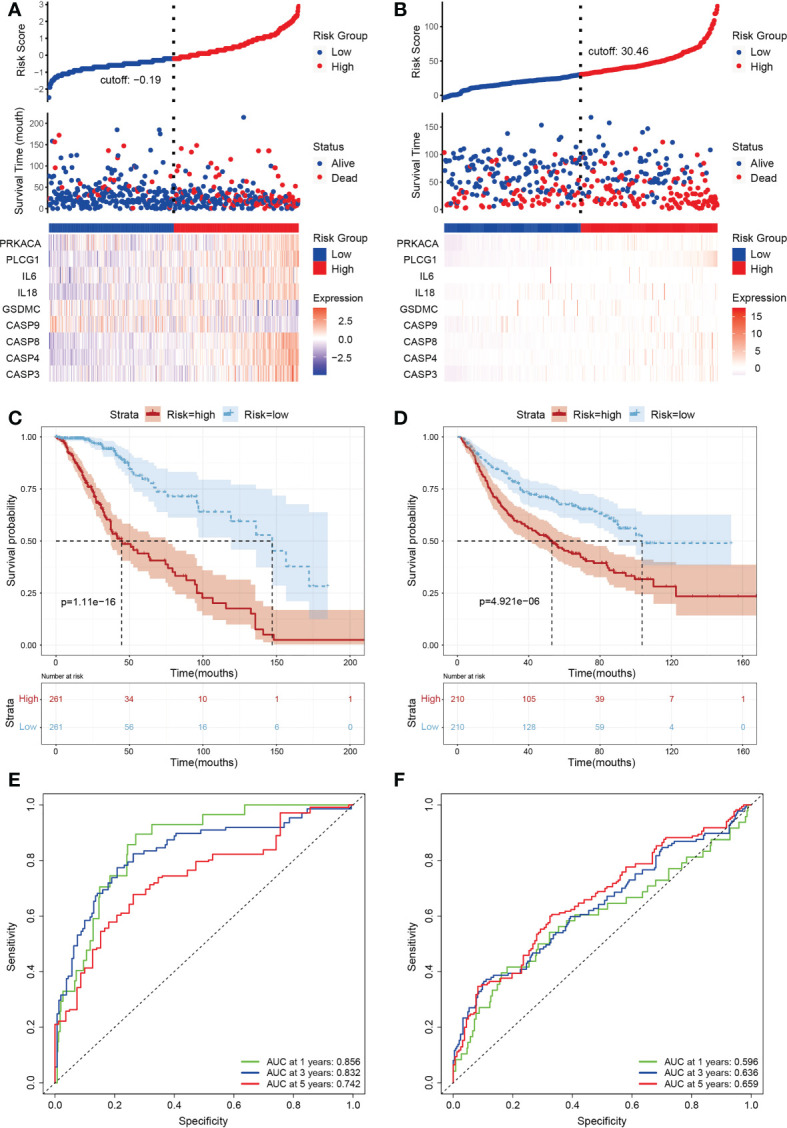
Subgroups of LGGs based on the construction of risk signature. **(A, B)** Distribution of risk score, differences in survival between the high- and low-risk groups and heatmap of the expression patterns of 9 pyroptosis-related genes in the training set **(A)** and testing set **(B)**. Red dots indicate dead, whereas blue dots indicate live. **(C, D)** Kaplan-Meier curve for the OS of LGG patients in the high- (n=194) and low-risk (n=194) groups in the TCGA training cohort **(C)** and the CGGA (n=420) testing cohort **(D)**. The red curve represents the high-risk group, and the blue curve represents the low-risk group. **(E, F)** Time-dependent ROC curve based on the prognostic model regarding OS and survival status in the TCGA training cohort **(E)** and the CGGA testing cohort **(F)**. Green, blue and red curves represent 1-year, 3-year and 5-year, respectively.

A total of 388 LGG patients were divided into high- (n=194) and low-risk (n=194) subgroups according to the median score calculated by the risk score formula(**Figures 3A, B**). Time-dependent Kaplan-Meier curves and receiver operating characteristic (ROC) curves were constructed to evaluate the sensitivity of the prognostic model (**Figures 3C, E**). The results showed differences in the survival curve between the low-risk group and the high-risk group (P<0.001) as the one year, three years, and five years AUC values were 0.856, 0.832 and 0.742, respectively. In addition, we matched 420 LGG patients in CGGA (Chinese Glioma Genome Atlas) as the validation set to further verify the model, resulting in one year, three years, and five years AUC values of 0.596, 0.636 and 0.659, respectively (**Figures 3B, D, F**).

### Clinical evaluation of the prognostic risk model

**Figure 4:**
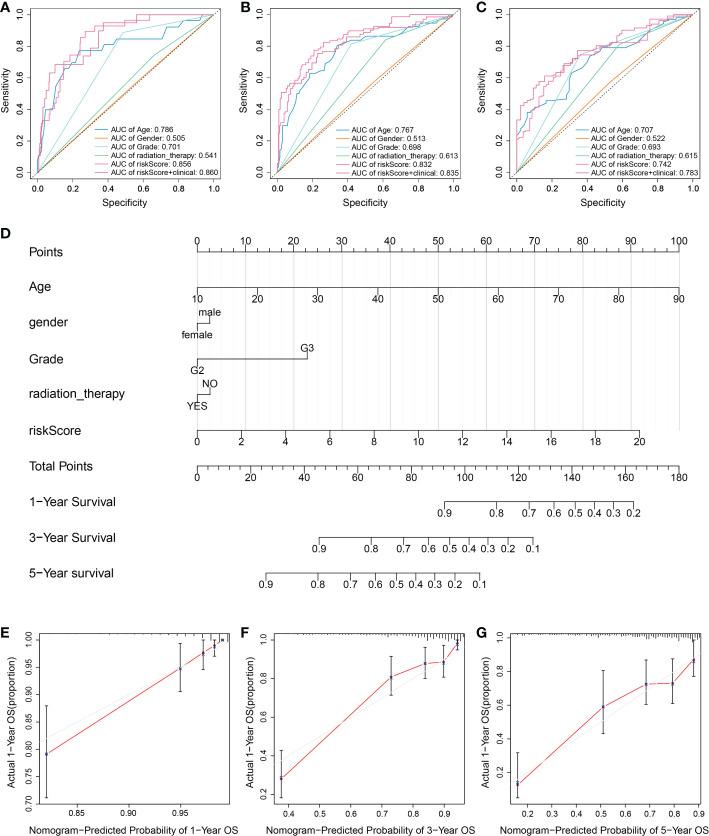
Assessment of the risk model combined with clinical factors. **(A-C)** Time-dependent ROC to evaluate prognostic power based on risk score and clinical factors at **(A)** 1-year, **(B)** 3-year, and **(C)** 5-year. Different colored curves represent different clinical factors. **(D)** A nomogram consisting of risk score and other clinical indicators for predicting 1-, 3-, and 5-year OS of primary LGG based on the TCGA cohort. **(E-G)** Nomogram calibration plots for predicting OS in the TCGA cohort at **(E)** 1-year, **(F)** 3-year, and **(G)** 5-year. The Red line indicates actual survival.

In order to verify practical value of prognostic model, univariate and multivariate COX analysis were used to evaluate whether the risk score of the model could also be an independent prognostic point compared with such clinical factors as grade, gender, age and radiation therapy. Univariate Cox regression analysis showed that risk scores were not associated with poor survival in both TCGA and CGGA groups of patients (**Figure S2A**). Interestingly, multivariate COX analysis showed similar trends to univariate COX regression results, suggesting that the prognosis of LGG is closely related with pyroptosis. According to ROC curve analysis, the AUC value remained at about 0.8 when combined with the risk score and clinical factors (**Figures 4A–C**). In addition, we used the TCGA and CGGA cohorts to analyze clinical factors and found that age, grade, and radiotherapy differed in the distribution of low- and high-risk categories (**Figures S2B, C**). We then integrated clinical variables and created a nomogram in the TCGA cohort (**Figure 4D**). The total score for each LGG patient was acquired by combining the scores of each prognostic standard in the nomogram. A higher total score would indicate patients with a worse prognosis. As a comparison, the predicted survival rate showed more consistency with the observed survival rate (**Figures 4E–G**).

### Functional and mutational analyses based on the risk model

**Figure 5:**
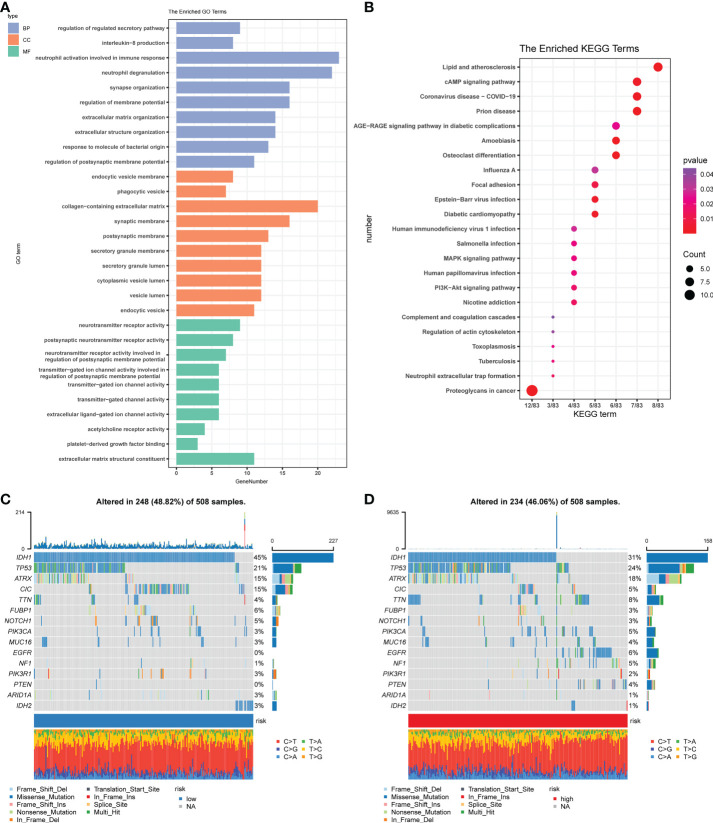
Functional and mutational analyses based on the risk model. **(A)** The enriched item in gene ontology analysis. Blue represents BP (biological process), orange represents CC (cellular component), and the green represents MF (molecular function). **(B)** The enriched item in Kyoto Encyclopedia of Genes and Genomes analysis. The color indicates the size of the P-value, and the size of the circle indicates the number of genes. **(C)** Mutation profile in the low-risk group. **(D)** Mutation profile in the low-risk group. The small figure above shows the TMB, whereas the number on the left shows the mutation frequency of each gene, and the figure on the right shows the proportion of each variant.

Subsequently, the DEGs were extracted and further explore the signal pathways related to different subgroups in the risk model. There are 181 differential genes were identified in the TCGA cohort, among which up-regulated of 109 genes and down-regulated of 72 genes in the high-risk group. Based on the differences between the risk subgroups, GO enrichment (**Figure 5A**) and KEGG (**Figure 5B**) pathway analysis was conducted. The results revealed the differential genes were mainly enriched in chemokine signaling pathways, cytokine-cytokine receptor interactions and immune response (**Figure 5**).

Combined with the results of gene enrichment in immune-related pathways, as suggested by functional analysis, we first compared the effects of cell mutations that tumors may induce. We used the ‘maftools’ R package on the high-risk group (**Figure 5C**) and the low-risk group (**Figure 5D**) to achieve a visual analysis of differences in the distribution of somatic mutations. Results showed the mutation rates among high-risk groups (46.06%) and low- (48.82%) to be relatively close.

### Immune activity and PCR analysis based on subgroups of the risk model

**Figure 6:**
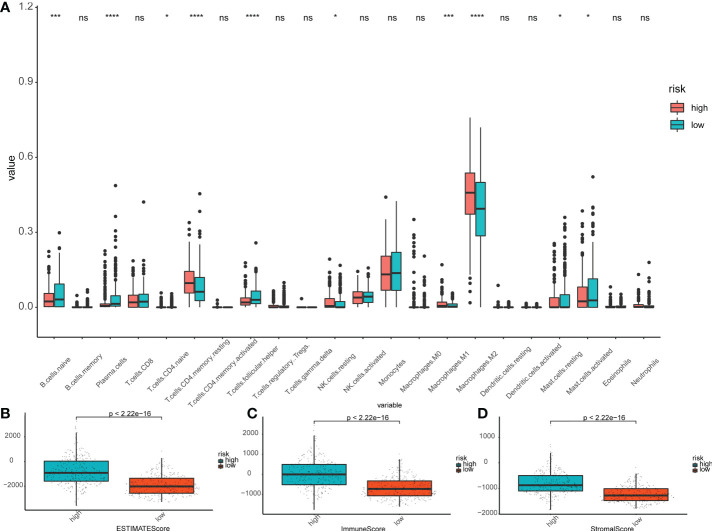
Immunoassay in the risk subgroups. **(A)** Boxplot showing the differential abundance of 22 infiltrating immune cells calculated by CIBERSORT between the high- and low-risk groups in LGG. ∗P< 0.05; ∗∗∗P< 0.001; ∗∗∗∗P< 0.0001; ns: not significant. **(B-D)** The higher expression level of Estimate Scores **(B)**, Immune Score **(C)**, and Stroma Score **(D)** correlated with the high-risk group. The green box shows the low-risk group, and the red box shows the high-risk group.

In order to further investigate the correlation among risk score of prognostic model and immune cell infiltration, ssGSEA module in the ‘GSVAR’ R package was used. This module quantifies the function of immune cells and the signal pathways in tumor samples. The scores for most immune cell types differed significantly between the low-risk and high-risk groups, which were confirmed in the validation set. The relative proportion of 22 types of immune cells associated with each LGG patient was calculated by using CIBERSOR and Estimate algorithms. The analysis of the correlation between the risk score and the degree of immune cell infiltration showed that many immune cells differed in the degree of infiltration between subgroups (**Figure 6A**); especially, plasma cells, TFH cells, and M2 macrophages were significantly upregulated (P<0.05). As a comparison, the Estimate results suggested the scores of patients were significantly higher in the high-risk group (**Figures 6B–D**).

**Figure 7:**
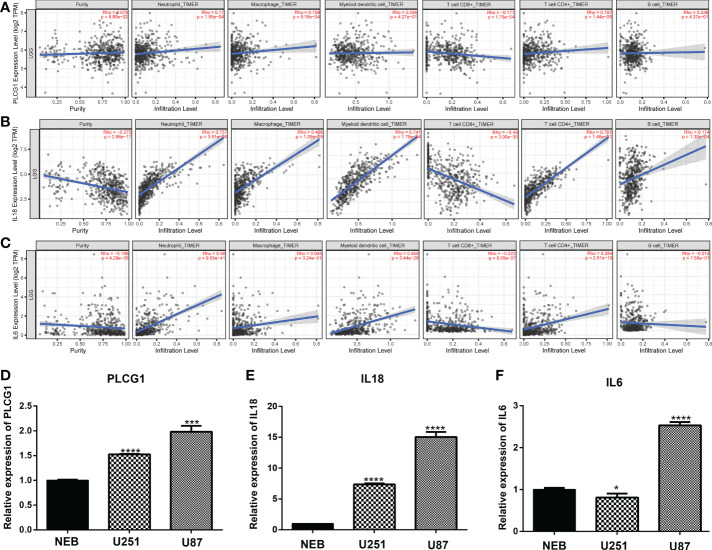
Expression of pyroptosis-related genes in immune cells and cell lines. **(A-C)** Association between the abundance of immune cells and the expression of three pyroptosis-related genes for PLCG1 **(A)**, IL18 **(B)**, and IL6 **(D)**. **(D-F)**. Quantitative Real-time PCR in cell lines for PLCG1 **(D)**, IL18 **(E)**, and IL6 **(F)**. ∗P< 0.05; ∗∗∗P< 0.001; ∗∗∗∗P< 0.0001.

We used TIMER database to analyses the relationship among 9 prognostic genes and immune cell abundance, and the results revealed that 9 genes of prediction model were all closely related to immune cell abundance (P<0.01, **Figures 7A-C** and **Figure S4**). To be more specific, the expression of PLCG1 was positively correlated with neutrophils, macrophages, myeloid dendritic cells, B cells and CD4+ T cells (**Figure 7A**), but negatively correlated with CD8+ T cells (**Figure 7A**). The expressions of CASP3 (**Figure S4A**), CASP4 (**Figure S4B**), CASP8 (**Figure S4C**), IL18 (**Figure 7B**), and IL6 (**Figure 7C**) were positively correlated with neutrophils. At the level of cellular immune infiltration, it is worth noting that CASP3 (**Figure S4A**) and CASP4 (**Figure S4B**) were positively correlated with macrophages and dendritic cells, but negatively correlated with CD4+ T cells and CD8+ T cells.

Furthermore, we randomly selected three pyroptosis-related genes (PLCG1, IL18, and IL6) in the prognostic model and confirmed the expression of three genes in cell lines through qPCR assays. Compared with the human normal glial cell line (HEB), PLCG1 (**Figure 7D**), IL18 (**Figure 7E**), and IL6 (**Figure 7F**) were highly expressed in cancer cell lines (U251 and U87). These three genes were therefore tightly associated with immunological activity in addition to being substantially expressed in cancer cell lines.

## Discussion

In this study, a prognostic model of 9 genes (CASP3, CASP4, CASP8, CASP9, GSDMC, IL18, IL6, PLCG1, and PRKACA) was constructed based on bioinformatics analysis with the survival data of LGG patients in the TCGA and CGGA cohorts and validated it by both clinical prognosis and correlation with tumor immunity. Although there are studies that have reported prognostic models related to LGG (38–42), these studies only used data from a single database - TCGA, thus the accuracy of the constructed models was limited. On the contrary, the present study used an external database (CGGA), which makes it more reliable for modeling. Furthermore, the constructed prognostic model was based on a newly discovered PCD, pyroptosis, making this current research fundamentally different from the others.

Initially, pyroptosis was thought to be PCD-activated by Caspase-1 only. However, Caspase-3/4/5/6/8/9/11 were successively identified to cause pyroptosis in different cell types; notably, Caspase-3 was found to be an essential molecule for membrane blistering and activation of apoptosis (43). Caspase-3 can cleave GSDME to make an alternative pathway to induce pyroptosis (44), which may be rapidly triggered by the Caspase-4/9 complex. Caspase-4 can also be turned on under infectious conditions to make a non-classical pyroptotic pathway by cleaving GASMD, along with caspase-5/11 (45, 46). Meanwhile, GASMD can stimulate macrophages to induce pyroptosis in a PLCG1 (phospholipase)-dependent pathway (47). Besides cleaving GSDMD directly, caspase-8 cleaves GSDMC when TNF-α is released to promote pyroptosis (48). By comparison, Xiao et al. (49) found that Caspase-3 influences dying glioma cells after chemotherapy by promoting tumor angiogenesis, leading to the recurrence of gliomas. Caspase-8 can also stimulate the NF-kB pathway and upregulate the secretion of IL-8, IL-1β, IL-6, VEGF and MCP-1, improving temozolomide resistance in gliomas (50). Caspase-9 had been reported to repair mitochondrial damage by maintaining autophagy, which then avoided influencing NLRP3 inflammasome to produce pyroptosis (51). These investigations all suggested that *via* inducing pyroptosis, the caspase family plays a dual role in gliomas.

Previous studies have revealed that proinflammatory effects of pyroptosis are closely related to the regulation of the tumor immune microenvironment (TIME) (52). In the TIME, tumor cells are protected by chemokines, cytokines, stromal cells, and metabolites that allow tumor cells to survive (53). Through damage-associated molecular patterns (DAMPs) released after cellular osmotic lysis, pyroptosis can reprogram TIME into an immune stimulatory state, so as to inhibit tumor cell growth and metastasis. Under the action of inflammatory factors, it can also promote the growth of tumor cells (54). Pyroptosis appeared to have a specific effect on TIME and facilitate immune surveillance (54). There has research has revealed NLRP3 inflammasome activation could affect pyroptosis or hyperactivity, resulting in a cascade of immune or inflammatory responses through the release of interleukins IL-β and IL-18, affecting antitumor immunity (55). In addition, IL-18 is upregulated in tumor-infiltrating lymphocytes (TILs) secreted by inflammasomes (56, 57). It has a protective pro-inflammatory effect, stimulating the generation of MDSCs to accelerate tumor progression (58). IL-6 performed an essential function in the regulation of macrophages and lymphocytes in the TME. Past studies reported that IL-6 directly influences the invasion of gliomas by activating the STAT3 pathway (59). PLCG1 and PRKACA are traditionally considered to be the executive molecules of apoptosis by signal transduction of phosphokinases. These molecules play regulatory roles in macrophage differentiation and inflammatory response to regulate the tumor microenvironment (60). Our prognostic model quantified the function of immune cells and signal pathways in tumor samples such that the scores for most immune cell types differed significantly between the low-risk and high-risk groups. In summary, our studies revealed different pyroptosis pathways (classical/non-classical/alternative/kinase-related), objectively reflecting the various factors influencing the development of LGG with a considerable reference value. Interestingly, Shao et al. (61) reported non-expression of GSDMD in the vast majority of tumor cells, suggesting that tumor cells can induce pyroptosis through more than one pathway, which is consistent with differential gene expression that forms the basis of our model. Though there is a slight lack of accuracy in judging the 5-year survival rate of LGGs, we still have a considerable advantage in the prognosis of LGG by considering the possibility of high-risk patients deteriorating into high-grade glioma within a short timeframe.

In order to analyze the immune infiltration of LGG tissue, our model compared different risk subgroups and found that naive B cells, plasma cells, TFH cells, and M2 macrophages were significantly upregulated in the high-risk group. Studies have shown accumulation of M2 macrophages can induce chronic inflammation, which is beneficial to the growth of invasive tumors. The intratumoral density of macrophages is highest in malignant gliomas, which is indicative of a negative correlation with the survival rate of patients (62). On the other hand, when combined with the analysis of the TIMER database, we further observed that the risk subgroups showed differences in the level of CD8+T cell infiltration. This is consistent with J. Robert Kane et al. whose study revealed that the accumulation of CD8+T cells in the TME indicates a better prognosis, while their absence is conducive to the growth of gliomas (63). In summary, we can speculate that pyroptosis regulates the TIME, which can be quantitated through our pyroptosis-related risk model, giving, in turn, a better prognostic picture than previous markers and models. Our further studies will elucidate the specific mechanisms.

Current tumor treatment strategies include, for example, immune checkpoint inhibitors, CAR-T, cytokines, virus lysis, and tumor vaccines, all widely used as representatives of immunotherapies in recent years (64). These methods have shown obvious carcinostatic effects in several tumors, but challenges remain in applying them to the treatment of glioma. One such challenge involves the physical blocking effect of the blood-brain barrier on drugs and cells. Another challenge arises from most gliomas that appear as a ‘cold tumor’ environment showing a higher level of immunosuppression, but fewer immune targets (65). Hung et al. addressed these challenges and found that large numbers of PD-L1 molecules enter the nucleus under tumor hypoxia. The complex of PD-L1 and phosphorylated STAT3 can mediate the expression of GSDMC and then induce tumor cell pyroptosis. In addition, pyroptosis combined with PD-L1 inhibitor can promote the conversion of ‘cold tumors’ into ‘hot tumors’. Liu et al. (66) reported that CAR-T cells activate the pyroptosis pathway of Caspase-3-GSDME by releasing GzmB, while co-culture experiments *in vitro* found that CAR-T can stimulate macrophages to release IL-6 and IL-1β by activating the Caspase-1-GSDMD pathway (67). Based on the studies above, our LGG prognostic model suggests a new approach to the treatment of gliomas by using it as a guide for the management of current mainstream immunotherapy.

There are still several limitations in our study. The analytical data were from public databases (TCGA and CGGA). To further confirmed the accuracy of pyroptosis-related gene prognostic model in predicting prognosis, the LGG tissues need to be collected and closely followed. Due to the lack of time and money for follow-up, the specific mechanism of pyroptosis-related genes has not been explored yet.

## Conclusion

Activation of inflammasome-related sensing proteins causes pyroptosis, a form of immune cell death. There are several that detect different substances. We identified differently expressed pyroptosis-related genes that may be involved in LGG. Pyroptosis may be an alternative therapeutic target since differentially expressed genes associated with it have significant predictive values for patient survival.

## Data availability statement

Publicly available datasets were analyzed in this study. This data can be found here: https://www.tcga.org/, http://www.cgga.org.cn/ and https://xenabrowser.net/.

## Author contributions

TT conceived, designed, and supervised the study. HL performed data analysis, arranged the figures and drafted the manuscript. All authors reviewed and approved the final manuscript. All authors contributed to the article and approved the submitted version.

## Supplementary material

Supplementary Figure 1 Expressions of pyroptosis-related genes in LGGs. **(A)** Heatmap of RNA expression levels of pyroptosis-related genes. Green represents a low expression level, and red represents a high expression level. **(B)** Pearson correlation analysis of the 33 pyroptosis-related genes in LGGs. Red indicates the degree of positive correlation, and blue indicates the degree of negative correlation.

Supplementary Figure 2 Analysis of clinical factors in subgroups. **(A)** Univariate analysis and multivariate analysis for hazard ratio values of risk score and clinical characters in the training set (CI, confidence interval). **(B-C)** Relationship between prognostic gene expression and clinical factors. Green represents a low expression level, and red represents a high expression level.

Supplementary Figure 3 Comparison of ssGSEA scores in high- and low-risk groups. **(A)** Training set. **(B)** Testing set. The green box shows the low-risk group, and the red box shows the high-risk group. ∗P< 0.05; ∗∗P< 0.01; ∗∗∗P< 0.001; ns: not significant.

Supplementary Figure 4 Association between the abundance of immune cells and the expression in LGGs. **(A)** CASP3. **(B)** CASP4. **(C)** CASP8. **(D)** CASP9. **(E)** GSDMC. **(F)** PRKACA.
