# Supplementary figures and images for: Prognosis and immune features of pyroptosis-related RNA patterns in low-grade glioma
Source: Front Oncol. 2022 Dec 20;12:1015850. doi: 10.3389/fonc.2022.1015850 (PMC9808047; doi:10.3389/fonc.2022.1015850)

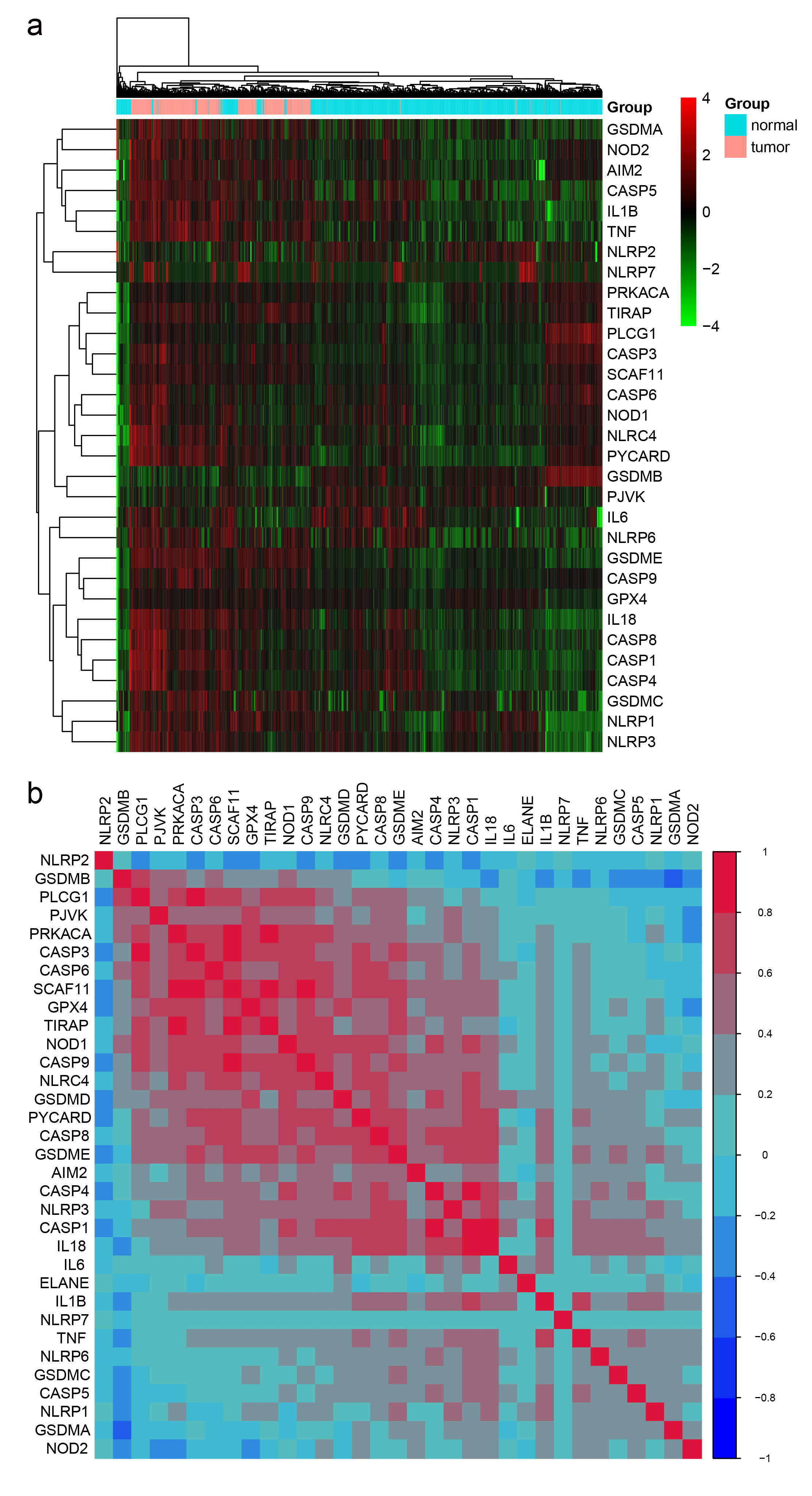

Supplement: Supplementary Figure 1 — Expressions of pyroptosis-related genes in LGGs. (A) Heatmap of RNA expression levels of pyroptosis-related genes. Green represents a low expression level, and red represents a high expression level. (B) Pearson correlation analysis of the 33 pyroptosis-related genes in LGGs. Red indicates the degree of positive correlation, and blue indicates the degree of negative correlation. [file Image_1.jpeg]

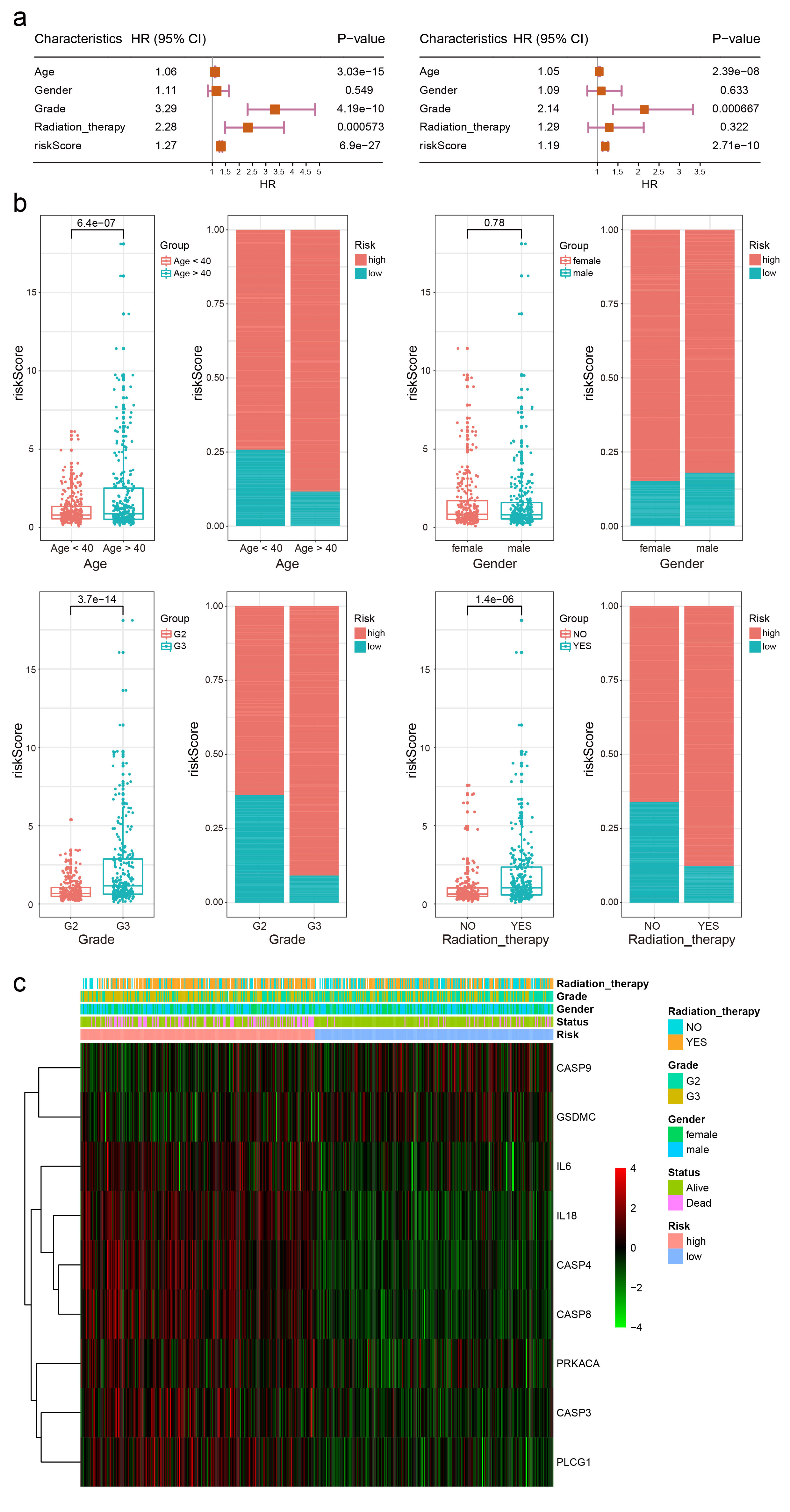

Supplement: Supplementary Figure 2 — Analysis of clinical factors in subgroups. (A) Univariate analysis and multivariate analysis for hazard ratio values of risk score and clinical characters in the training set (CI, confidence interval). (B-C) Relationship between prognostic gene expression and clinical factors. Green represents a low expression level, and red represents a high expression level. [file Image_2.jpeg]

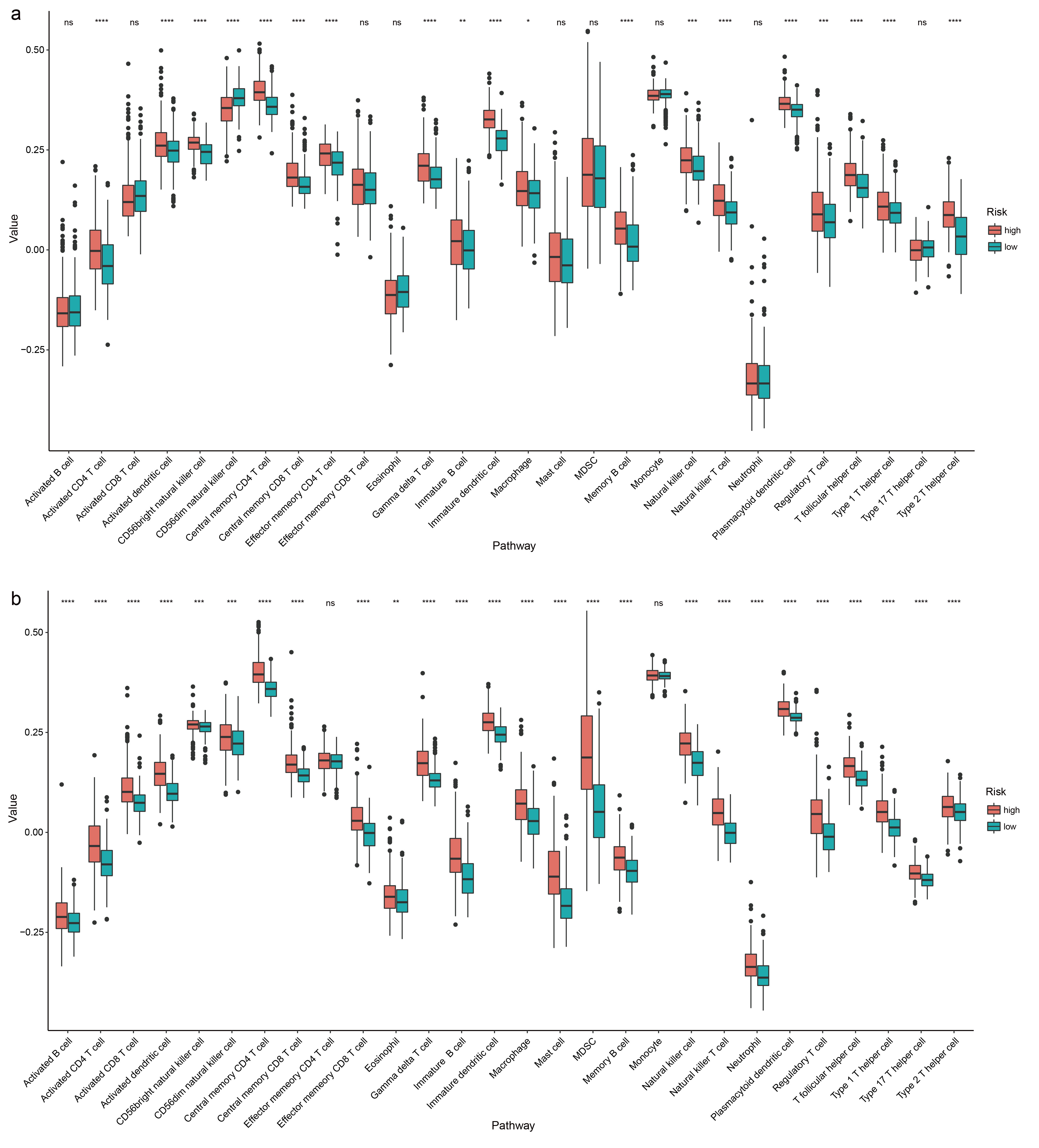

Supplement: Supplementary Figure 3 — Comparison of ssGSEA scores in high- and low-risk groups. (A) Training set. (B) Testing set. The green box shows the low-risk group, and the red box shows the high-risk group. ∗P< 0.05; ∗∗P< 0.01; ∗∗∗P< 0.001; ns: not significant. [file Image_3.jpeg]

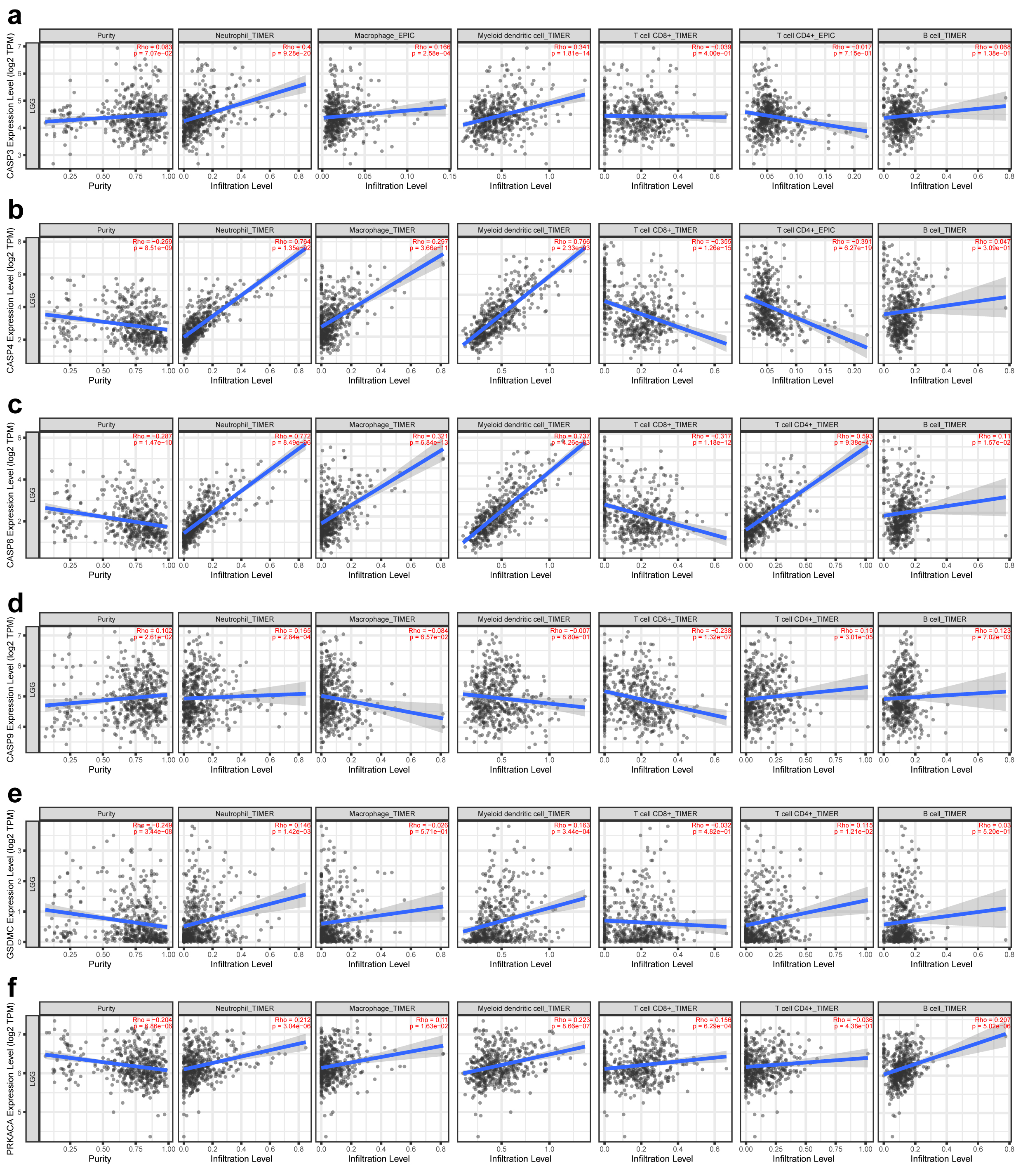

Supplement: Supplementary Figure 4 — Association between the abundance of immune cells and the expression in LGGs. (A) CASP3. (B) CASP4. (C) CASP8. (D) CASP9. (E) GSDMC. (F) PRKACA. [file Image_4.jpeg]
